# Society Meetings

**Published:** 1897-10

**Authors:** 


### Society Meetings.

The Buffalo Academy of Medicine held meetings during the month of September as follows :

*Section on Medicine.*—Tuesday evening, September 14th, program : Medico-legal aspects of cases of poisoning; with especial reference to restriction of sale of poisons, by Dr. A. L. Benedict and Hon. Arthur W. Hickman.

*Section on Pathology.*—Tuesday evening, September 21, program : Some observations on the physiology of the stomach ; Report of X-ray exhibition, by Dr. A. L. Benedict ; Abnormal cell development in plants and animals :

a study in comparative pathology, by Miss Mary Forster, Newnham College, Eng.

*Section on Medicine.*—Tuesday evening, September 28th, program: Tuberculosis, by Dr. Wm. H. Bergtold, professor of pathology, University of Denver, Colorado; discussion to be opened by Drs. F. J. Thornbury and S. S. Greene.

---

THE American Academy of Railway Surgeons will hold its fourth annual meeting at the Auditorium Hotel, Chicago, Wednesday, Thursday and Friday, October 6, 7 and 8, 1897, under the presidency of Dr. L. E. Leman, of Denver. The preliminary program lately issued contains an interesting list of papers and discussions. Among those announced to participate are Drs. E. M. Dooley and C. M. Daniels, of Buffalo.

---

THE New York State Association of Railway Surgeons will hold its seventh annual meeting at the Academy of Medicine, New York City, on Tuesday, November 16, 1897, under the presidency of Dr. J. Frank Valentine, chief surgeon of the Long Island Railway.

---

THE American Electro-therapeutic Association met at Harrisburg, September 21, 22 and 23, 1897. Dr. L. A. Weigel, of Rochester, read a paper upon Electricity in orthopedic practice. Dr. Charles B. Dickson, of Toronto, was elected president for the ensuing year and the next meeting was appointed to be held at Buffalo.

---

THE Roswell Park Medical Club held its annual meeting at the office of Dr. Thomas Bagley, 1344 West avenue, Buffalo, on Monday evening, September 13, 1897. The following-named officers were elected for the ensuing year: president, Dr. William Meisburger; vice-president, secretary and treasurer, Dr. William Dignen. After the business was transacted Dr. Bagley entertained the club.

---

THE Medical Association of Central New York will hold its thirtieth annual meeting at the Ellicott Club, Buffalo, N. Y., Tuesday, October 19, 1897. A preliminary program of the papers will soon be published, hence those who desire to read should send titles to the secretary at once. The Transactions of this society are published by the JOURNAL and all papers read must be contributed exclusively to it, if it is desired to have them appear in the Transactions.

The association will hold a morning session from 10 to 1 and an afternoon session from 2 to 6 o'clock.

Those who desire to join should obtain delegate papers from the president and secretary of the Medical Society of the County of Erie and present them to the secretary of the association, Dr. C. A. Vander Beek, 113 North street, Rochester, N. Y.

